# Supplementary material for: Automated evaluation of colon capsule endoscopic severity of ulcerative colitis using ResNet50
Source: PLoS One. 2022 Jun 10;17(6):e0269728. doi: 10.1371/journal.pone.0269728 (PMC9187078; doi:10.1371/journal.pone.0269728)
Supplement: Attachment — Submitted filename: ethics approval 0126 translated in English.docx [file pone.0269728.s004.docx]

July 4, 2017

Dean of Hirosaki University of Graduate School of Medicine

Koichi Wakabayashi

(Report)

“The usefulness of pan-enteric survey using colon capsule endoscopy, Total Observation from intestine To Anal Lesion: TOTAL study (rapid review #2017-1046)” was applied for ethics review by Hiroto Hiraga (Odate/Kitaakita Regional Medical Promotion Course, Research associate), and the ethics committee of Hiroaki University Graduate School of Medicine discussed carefully, and approved the application.

The ethics committee of Hiroaki University Graduate School of Medicine

Sadatomo Tasaka

Tadaatsu Imaizumi

Manabu Murakami

Hiroya Mizukami

Kazuhiko Nakamura

Yoshihiro Sasaki

Makoto Daimon

Ikuko Kakizaki

Koh Kudo

Kumiko Hanada

Kiyoshi Hirano

Yuka Shinozaki

Hiroo Nakabayashi

Atsushi Nagasawa
