# Supplementary material for: Automated evaluation of colon capsule endoscopic severity of ulcerative colitis using ResNet50
Source: PLoS One. 2022 Jun 10;17(6):e0269728. doi: 10.1371/journal.pone.0269728 (PMC9187078; doi:10.1371/journal.pone.0269728)
Supplement: Attachment — Submitted filename: Response to Reviewers.docx [file pone.0269728.s005.docx]

Dear Dr. Chenette, Dr.Mizoguchi:

Thank you for giving me the opportunity to submit a revised draft of my manuscript titled **“Automated evaluation of colon capsule endoscopic severity of ulcerative colitis using ResNet50**” with *PLOS ONE*. We appreciate the time and effort that you and the reviewers have dedicated to providing your valuable feedback on my manuscript. We are grateful to the reviewers for their insightful comments on our paper. We have been able to incorporate changes to reflect most of the suggestions provided by the reviewers. We have indicated the changes within the manuscript in red, light blue, and green font colors. Here is a point-by-point response to the reviewers’ comments and concerns.

**・Response to journal requirements:** Thank you for your valid comments. I agree with all four of the suggestions and have incorporated them to the revised version of the manuscript (green color).

1. Yes

2. Yes

3. Yes

4. I agree with you. I have inserted the following citation in the revised manuscript.

-https://www.sciencedirect.com/science/article/abs/pii/S0010482520302857?via%3Dihub

Lastly, according to the changes mentioned above, I have deleted some redundant sentences.

The deleted sentences were on Page 3, Lines 54-55 of the original version of the manuscript.

**・Response to Reviewer #1:** Thank you for giving me good advice. I agree with both the suggestions and have revised the manuscript accordingly (changes are indicated in red font color).

1. *As authors stated, the large number of images (52.4%) have been classified as "inadequate quality for analysis". Do authors think that this examination is intrinsically reliable and reproducible? Please describe about it.*

**Response:** There were images that were taken randomly along the lesions that were of poor quality. We do not think that the large number of unanalyzed images, because of inadequate quality, introduced any kind of bias that resulted in imprecise results in this study. The UC lesions were missed rarely by capsule endoscopy, because of their diffuse distributions. This characteristic of the disease has potentially contributed to avoiding the bias. We have incorporated this point in the limitations of the revised manuscript (lines 312–318 of the revised manuscript).

2. *How much amount of liquid preparation did the patients have in MOVIPREP method for colon capsule? Is it tolerable and ethically acceptable for UC patients with severe MES 3? In severe condition of UC, most clinicians evaluate the severity by sigmoidoscopy without oral liquid preparation which may cause flare-up. Please describe about it.*

**Response:** We excluded UC patients with severe conditions—as determined by the questionnaire—from this study. Additionally, it is not possible to predict very severe lesions in patients without severe subjective symptoms prior to investigations. No severe subjective symptoms developed in the participants with MES 3 at the time of the examination. Nevertheless, we know that if UC patients with severe subjective symptoms had undergone the MOVIPREP regimen, it would have been intolerable and unacceptable to them. Based on the aforementioned reasons, we think that the methods of the study were tolerable and ethically correct for the patients. Although, we do acknowledge that liquid preparation could have caused flare-ups among the patients with severe lesions, as the reviewer pointed out. Accordingly, we have added this limitation to the revised manuscript (lines 308–312 of the revised manuscript).

I believe that incorporating your advice into the revised version has made the manuscript better. Thank you once again.

**・Response to Reviewer #2:** Thank you for the excellent advice. I agree with all four of your suggestions and have revised the manuscript accordingly (revisions are indicated in light blue color font).

1. *Bleeding is very important in assessing the severity of the disease, but how is it assessed in this study? Please add this to your manuscript on the evaluation method.*

**Response:** I agree with your suggestion. Bleeding is very important in assessing the severity of the disease, especially in patients with severe UC. However, we excluded UC patients with severe conditions from this study, because MOVIPREP method would have been intolerable and it would have been ethically wrong to cause harm. We have added the exclusion criterion aforementioned in the method section of the revised manuscript (lines 134–136 of the revised manuscript).

2. *What was the reason you did not evaluate it in the UCEIS? Also, if you believe that MES is sufficient for evaluation, please explain the reason.*

**Response:** Although UCEIS is recognized to be a more accurate assessment of mucosal severity in patients with UC as compared to MES, UCEIS is designed to evaluate the severity with a single image. In contrast, the advantage of capsule endoscopy is to obtain and evaluate serial images of the whole colon. Moreover, our procedure can construct a topographic map of the severity. To augment these assets, we selected MES and not UCEIS to evaluate the severity. We have added the reasons for adopting MES in the method section of the revised manuscript (lines 160–165 of the revised manuscript).

3. *For Figure 5, I think it would be easier to visualize the activity of the entire colon if there were 3D graphs. If possible, please considering add those images.*

**Response:** We converted Figure 5, which was composed of two-dimension graphs, into three-dimension graphs, but, unfortunately, they were not easy to understand and did not add new information for readers. If the reviewer can please suggest to us what is required more specifically, we will try put it into practice.

4. *How are UC-complicated intestinal lesions, such as cytomegalovirus enteritis and UCAN, diagnosed? Or are they excluded? Please add this to your manuscript.*

**Response:** Small trimmed patch images could not be evaluated among those obtained by capsule endoscopy, so our diagnoses did not include UC-complicated intestinal lesions, such as cytomegalovirus enteritis and UCAN. We recognized it as a limitation of this study, and have added it to the limitation subsection of the revised manuscript (lines 318–321 of the revised manuscript).

I believe that incorporating your advice in the revised version has made the manuscript better. Thank you once again.
